# A microglial activity state biomarker panel differentiates FTD-granulin and Alzheimer’s disease patients from controls

**DOI:** 10.1186/s13024-023-00657-w

**Published:** 2023-09-29

**Authors:** Ida Pesämaa, Stephan A. Müller, Sophie Robinson, Alana Darcher, Dominik Paquet, Henrik Zetterberg, Stefan F. Lichtenthaler, Christian Haass

**Affiliations:** 1 German Center for Neurodegenerative Diseases (DZNE) Munich, Munich, Germany; 2 Graduate School of Systemic Neurosciences (GSN), Ludwig-Maximilians-University Munich, Munich, Germany; 3 Department of Psychiatry & Neurochemistry, Institute of Neuroscience & Physiology, the Sahlgrenska Academy at the University of Gothenburg, Mölndal, Sweden; 4 Institute for Stroke and Dementia Research, University Hospital Munich, Ludwig-Maximilians- University Munich, Munich, Germany; 5 Epileptology, University Hospital Bonn, Bonn, Germany; 6 Neuroproteomics, School of Medicine, Klinikum Rechts der Isar, Technical University of Munich, Munich, Germany; 7 Munich Cluster for Systems Neurology (Synergy), Munich, Germany; 8 Clinical Neurochemistry Laboratory, Institute of Neuroscience and Physiology, Sahlgrenska University Hospital, Mölndal, Sweden; 9 Department of Neurodegenerative Disease, UCL Institute of Neurology, Queen Square, London, UK; 10 UK Dementia Research Institute at UCL, London, UK; 11 Hong Kong Center for Neurodegenerative Diseases, Clear Water Bay, Hong Kong, China; 12 Wisconsin Alzheimer’s Disease Research Center, School of Medicine and Public Health, University of Wisconsin, University of Wisconsin-Madison, Madison, WI USA; 13 Biomedical Centre (BMC), Faculty of Medicine, Ludwig-Maximilians-University Munich, Munich, Germany

**Keywords:** Alzheimer, Biomarker, Frontotemporal Dementia, Microglial activity, Patient stratification, Therapeutic modulation

## Abstract

**Background:**

With the emergence of microglia-modulating therapies there is an urgent need for reliable biomarkers to evaluate microglial activation states.

**Methods:**

Using mouse models and human induced pluripotent stem cell-derived microglia (hiMGL), genetically modified to yield the most opposite homeostatic (*TREM2-*knockout) and disease-associated (*GRN*-knockout) states, we identified microglia activity-dependent markers. Non-targeted mass spectrometry was used to identify proteomic changes in microglia and cerebrospinal fluid (CSF) of *Grn*- and *Trem2*-knockout mice. Additionally, we analyzed the proteome of *GRN*- and *TREM2*-knockout hiMGL and their conditioned media. Candidate marker proteins were tested in two independent patient cohorts, the ALLFTD cohort (*GRN* mutation carriers versus non-carriers), as well as the proteomic data set available from the EMIF-AD MBD study.

**Results:**

We identified proteomic changes between the opposite activation states in mouse microglia and CSF, as well as in hiMGL cell lysates and conditioned media. For further verification, we analyzed the CSF proteome of heterozygous *GRN* mutation carriers suffering from frontotemporal dementia (FTD). We identified a panel of six proteins (FABP3, MDH1, GDI1, CAPG, CD44, GPNMB) as potential indicators for microglial activation. Moreover, we confirmed three of these proteins (FABP3, GDI1, MDH1) to be significantly elevated in the CSF of Alzheimer’s (AD) patients. Remarkably, each of these markers differentiated amyloid-positive cases with mild cognitive impairment (MCI) from amyloid-negative individuals.

**Conclusions:**

The identified candidate proteins reflect microglia activity and may be relevant for monitoring the microglial response in clinical practice and clinical trials modulating microglial activity and amyloid deposition. Moreover, the finding that three of these markers differentiate amyloid-positive from amyloid-negative MCI cases in the AD cohort suggests that these proteins associate with a very early immune response to seeded amyloid. This is consistent with our previous findings in the Dominantly Inherited Alzheimer’s Disease Network (DIAN) cohort, where soluble TREM2 increases as early as 21 years before symptom onset. Moreover, in mouse models for amyloidogenesis, seeding of amyloid is limited by physiologically active microglia further supporting their early protective role. The biological functions of some of our main candidates (FABP3, CD44, GPNMB) also further emphasize that lipid dysmetabolism may be a common feature of neurodegenerative disorders.

**Supplementary Information:**

The online version contains supplementary material available at 10.1186/s13024-023-00657-w.

## Background

During the last decade, genome-wide association studies (GWAS) and whole genome sequencing have identified microglial expressed risk variants for late onset Alzheimer’s disease (LOAD) [1]. Triggering receptor expressed on myeloid cells 2 (TREM2) plays a central role in the coordinated switch of microglial activity states upon pathological challenges, and disease risk variants lose their physiologically required activity [2, 3]. These findings allowed to further elucidate the role of microglia in neurodegenerative disease and initiated the development of microglia modulating therapies [3]. TREM2 is expressed as a type 1 protein, which is targeted to the cell surface together with its co-receptor DNAX activation protein of 12 kDa (DAP12) [4]. On the cell surface TREM2 is proteolytically cleaved to liberate soluble TREM2 (sTREM2). Brain derived sTREM2 is found in biological fluids, such as blood and cerebrospinal fluid (CSF) [5]. Cleavage of TREM2 terminates its cell-autonomous signaling functions [5]. Since predominantly cell surface TREM2 is cleaved, sTREM2 concentrations in biological fluids are a proxy of signaling-competent full-length TREM2 [6]. sTREM2 levels increase immediately after the initial seeding process when amyloid fibers precipitate and start to form amyloid plaques [7]. This occurs extremely early during disease progression. In patients with familial AD, sTREM2 increases up to 21 years before disease onset [7]. Thus, the early TREM2 response of microglia in AD is driven by amyloid precipitation and is apparently a defensive response to a pathological challenge. In fact, seeding of amyloid pathology is boosted in the absence of functional TREM2 [8]. Moreover, increased sTREM2 levels are linked to reduced brain shrinkage and a better cognitive outcome [9]. Based on these findings, microglia appear to have protective functions at least early during amyloidogenesis. To boost such protective microglial functions, monoclonal antibodies were developed to selectively stabilize and cross-link cell surface TREM2 with the goal to enhance its protective signaling activities [3, 10]. First clinical trials are already on the way [3]. TREM2 boosting therapies rely on the development of biomarkers, which allow to monitor target engagement, efficacy of treatment and even stratification of patients with low or high microglial response. Therefore, biomarkers are required, which allow the determination of microglial responses to pathological challenges in human biofluids.

To identify such markers, we made use of models lacking either *TREM2* or the lysosomal protein progranulin (*GRN*) providing us with two completely opposite microglial activation phenotypes. In *TREM2* knock-out (ko) models, microglia are locked in a homeostatic state, while microglia from *GRN* ko models are hyperactivated [11, 12]. These models therefore capture the two most opposite activity states of microglia and provide optimal models to identify proteins, which reflect either resting or responsive microglial activities. Using mass spectrometry (MS)-based proteomics, we analyzed the proteome of microglia and CSF obtained from *Trem2* ko and *Grn* ko mice as well as cell lysates and media from *TREM2* and *GRN* KO human-induced pluripotent stem cell (iPSC)-derived microglia (hiMGL). Hyperactivated microglia were also reported in patients suffering from frontotemporal dementia (FTD) caused by *GRN* haploinsufficiency (FTD-GRN) [13, 14]. As a first translational approach, we therefore analyzed the CSF proteome of FTD-GRN patients versus healthy non-carriers. This allowed the identification of six proteins as our top biomarker hits, which were consistently increased in hiMGL and in the CSF of *GRN* carriers. We confirmed our potential candidates in AD patients, for which we made use of proteomic data based on human CSF collected as part of the European Medical Information Framework (EMIF)-AD Multimodal Biomarker Discovery (MBD) study [15]. Strikingly, three of the top candidate activity state markers were capable to differentiate amyloid-positive from amyloid-negative individuals diagnosed with mild cognitive impairment (MCI) independently from each other.

## Methods

### Study aim and study design

The aim of this study was to identify biomarker candidates for the evaluation of microglial activation states in humans. The design of this study is rather unique for a biomarker discovery study, starting with in vivo (mouse) and in vitro (hiMGL) models for microglial activation with a final confirmation of biomarker candidates in human CSF. The main purpose of this study design was to make use of the data generated from microglial activation models to assure that the selected biomarker candidates, detectable in human CSF, indeed reflect the microglial activation state.

### Mice

All mice were housed in standard sized individually ventilated cages (IVC), with enriched environment and ad libitum access to food and water. Mice were maintained in a specific pathogen-free facility with a 12-hour light/dark cycle. All experiments and handling of mice was performed in compliance with the German animal welfare law and with approval from the Government of Upper Bavaria. CSF was sampled under the animal license: ROB 55.2–2532.Vet_02-15-69. Afterwards, mice were sacrificed by cervical dislocation followed by brain harvest. CSF and brain tissue were collected from mice of the following mouse strains: C57BL/6J, C57BL/6J *Grn* knockout [16], and C57BL/6J *Trem2* knockout [17]. The genotyping of these mice was performed as previously described [13, 16, 17]. Both male and female mice were used for the experiments.

### Mouse CSF sampling

Mice were deeply anesthetized using a mix of medetomidine [0.5 mg/kg], midazolam [5 mg/kg], and fentanyl [0.05 mg/kg] (MMF). The MMF-mix was dosed according to bodyweight of each mouse and administrated via intraperitoneal injection. CSF was collected from the cisterna magna by a single stereotactic puncture of the dura using a glass capillary, following a previously described procedure [18]. The collected CSF samples were centrifuged at 2000 x g for 10 min and visually controlled for blood contamination.

### Microglia isolation from mouse brain – MACS

Immediately after CSF sampling, brains were harvested and microglia were isolated using the Magnetic-Activated Cell Sorting (MACS) technique, following the manufactures instructions (Neural Tissue Dissociation Kit (P), MACS Miltenyi Biotec), with slight modifications similarly to a previously published protocol [19]. In brief, cerebellum, olfactory bulb, and meninges were removed, and each hemisphere was cut into pieces. Dissected brains were homogenized using an automatic (gentleMACS Dissociator) and enzymatic (Enzyme A (10 µl) + Enzyme P (50 µl)) dissociation process. The homogenates were rinsed through a cell strainer (40 μm, Falcon). Filtered samples were pelleted, washed and resuspended in Hanks’ buffered salt solution (HBSS) supplemented with 7mM HEPES. After washing, cells were magnetically labeled with CD11b (Microglia) Microbeads (Miltenyi Biotec) and diluted in 90 µl MACS buffer (PBS supplemented with 5% BSA). The magnetically labeled cell suspension was loaded onto a MACS LS-column, washed with MACS buffer, and eluted into a Protein LoBind tube. The eluted microglia fraction was pelleted and washed with HBSS to remove any remaining BSA, before all liquid was aspirated and the remaining cell pellet was snap-frozen and stored at -80 °C.

### Human induced pluripotent stem cell (iPSC) culture, CRISPR genome editing and differentiation of iPSC-derived microglia (hiMGL)

All experiments including iPSCs were performed in compliance with all applicable guidelines and regulations. iPSC culture of the female iPSC line A18944 (ThermoFisher, #A18945) based on E8 Flex/VTN (ThermoFisher, #A2858501 and #A14700), CRISPR/Cas9-based genome editing, iPSC quality control measures were performed as described previously for the *GRN* ko line [20], with the exception that NEB enzyme SfaNI was used for the analysis with Restriction fragment length polymorphism technique (RFLP) for the *TREM2* ko line, while the MwoI enzyme was used for the *GRN* ko line. We further explain parameters for the generation of *TREM2* ko iPSCs in the Appendix Figure S1. Differentiation of iPSCs to hiMGL was also performed as described [20]. In brief, the iPSCs were differentiated into hematopoietc progenitor cells for 12 days using a HPC differentiation kit (Stemcell Technologies) and then further into hiMGL, with cytokines M-CSF, TGF-β, and IL-34, added fresh. As shown previously hiMGL express typical microglial markers, including TMEM119 [21].

### Harvesting of hiMGL pellets and conditioned media

Throughout the procedure, cells and PBS were kept on ice. In total, two 6-well plates from each experimental group (WT, *GRN* ko, and *TREM2* ko) were used. The cells were carefully dissociated from the plate using a cell scraper, washed with their media, and subsequently transferred to centrifugation tubes (15 ml), pooled pairwise, yielding n = 6 for each experimental group. The tubes were centrifuged at 3000 x g for 6 min at 4 °C and the conditioned media was transferred to a Protein LoBind tube (Eppendorf) (media fraction). Each well of the plates was washed with 0.5 ml PBS and pooled following the same order as the pooling of the collected cells in the same 15 ml centrifugation tube and the pellet resuspended, resulting in a total volume of 1 ml and n = 6 per experimental group. The 1 ml of PBS with the resuspended cells was transferred to a 1.5 ml Protein LoBind tube (Eppendorf), centrifuged at 3000 x g for 6 min at 4 °C, and PBS aspirated, leaving the pellet. The conditioned media was aliquoted and stored at -80 °C (media fraction). Each cell pellet was stored at -80 °C (microglia fraction).

### Human CSF samples

In total, CSF of 12 symptomatic FTD heterozygous *GRN* mutation carriers (FTD-GRN) and 12 non-symptomatic non-carriers (CON) were analyzed. Data from one *GRN* mutation carrier was excluded as this participant was not fulfilling the criteria of symptomatic FTD, resulting in a comparison of 11 FTD-GRN and 12 CON. All participants were enrolled in ARTFL (U54NS092089) or LEFFTDS (U01AG045390), together ALLFTD, which is a North American research consortium to study sporadic and familiar FTD. ALLFTD has 27 sites in North America. Participants are between the ages of 18–85. Inclusion criteria for ALLFTD are clinical diagnosis of an FTD syndrome, or being a member of a family with a known FTD-associated genetic mutation or a strong family history (modified Goldman score of 1 or 2) of FTD syndromes. For the study presented here, the group defined as FTD-GRN was defined as symptomatic GRN-mutation carriers. All CSF from the ALLFTD consortium were approved and provided by the National Centralized Repository for Alzheimer’s Disease and Related Dementias (NCRAD). CSF obtained from ALLFTD was collected following NCRAD standard operating procedure. In brief, CSF was collected in the morning between 8 am – 10 am, preferably fasted or following a low-fat diet. Lumbar puncture was performed in a sterile field using an atraumatic technique. Within 15 min of collection, CSF samples are centrifuged at 2000 x g for 10 min at room temperature. Post centrifugation CSF is aliquoted into pre-cooled aliquot tubes and within 60 min of CSF collection aliquots are snap frozen on dry ice and stored at -80 °C. Data from the European Medical Information Framework Alzheimer’s Disease Multimodal Biomarker Discovery (EMIF-AD MBD) cohort are publicly available [15, 22]. Proteomic data from 478 participants were filtered for FABP3, MDH1, GDI1, CAPG, CD44, GPNMB. The abundances of each of the selected proteins were compared across the cohort groups which were defined according to diagnosis as cognitively normal controls (CNC, n = 126), subjective cognitive impairment (SCI, n = 61), mild cognitive impairment (MCI, n = 198), and Alzheimer’s disease (AD, n = 93). The presence or absence of amyloid pathology for the participants of the EMIF-AD MBD cohort was previously assessed by others based on levels of CSF amyloid-β_1−42_[22], which allowed for further stratification of the cohort groups as amyloid-positive (A^+^) and amyloid-negative (A^−^). For correlation analysis CSF levels of TREM2, GFAP and CHI3L1, as included in the publicly available data [15], were included. The ALLFTD and EMIF-AD MBD are both cross-sectional observational studies.

### Sample preparation for mass spectrometry

In total, 14 sets of samples have been collected from mice, hiMGL and participants of the ALLFTD study. Samples were prepared and analyzed with liquid chromatography tandem mass spectrometry (LC-MS/MS) using a label-free quantification (LFQ) method. The mouse cohort consists out of three mouse lines: Wt, *Grn* ko, and *Trem2* ko – all sacrificed when 12 months old. In total, we analyzed CSF from 14 mice (n(Wt) = 6; n(*Grn* ko) = 4; n(*Trem2* ko) = 4) and microglia from 19 mice (n(Wt) = 7; n(*Grn* ko) = 4; n(*Trem2* ko) = 8). The hiMGL samples included both cell lysates and conditioned media, each collected from three different iPSC lines: WT, *GRN* ko, and *TREM2* ko, with a sample size of n = 6 for each sample type and line. For human CSF, the CSF of 11 symptomatic FTD heterozygous *GRN* mutation carriers (FTD-GRN) and 12 non-symptomatic non-carriers (CON) was analyzed. All samples were prepared and analyzed together according to sample type; mouse CSF, mouse microglia, hiMGL media, hiMGL cell lysates, and hCSF. Mouse CSF was prepared using in-solution digestion. Mouse microglia, and hiMGL media was prepared using filter aided sample preparation (FASP) [23], while hiMGL cell lysates and hCSF was prepared using Single-pot, solid-phase-enhanced sample preparation (SP3) [24].

### In-solution digestion

From each sample of mouse CSF, a volume of 5 µl was used for in-solution digestion in 0.1% (w/v) sodium deoxycholate according to a previously published protocol [25]. Peptides were purified by stop and go extraction (STAGE) with C18-packed pipetting tips [26] and dried using vacuum centrifugation.

### Single-pot, solid-phase-enhanced sample preparation - SP3

15 µl of human CSF was mixed with 15 µl modified STET lysis buffer (150 mM NaCl, 2 mM EDTA pH 8.0, 50 mM Tris pH 7.5, 2% Triton X-100) to inactivate potential viruses. For hiMGL, cell pellets were lysed on ice in STET lysis buffer supplemented with protease inhibitors (Protease Inhibitor Cocktail, Sigma Aldrich, Product P8340). The SP3 sample preparation was performed according to a previously published protocol [27] using 70% (v/v) acetonitrile for protein binding and four washing steps with 80% (v/v) ethanol. After the sequential digestion with LysC and trypsin (Promega, each protease to protein ratio 1:80), beads were retained with a magnetic rack and samples were filtered with Costar Spin-X spin filters (0.22 μm cut-off) to remove remaining beads, and samples were dried by vacuum centrifugation.

### Sample preparation for mass spectrometry – FASP

Mouse microglia and hiMGL conditioned media were digested using filter aided sample preparation (FASP) as previously described [23]. For microglia lysates Vivacon spin filters (Sartorius) with 30 kDa cut-off were used, whereas conditioned media samples (500 µl) were concentrated on Vivacon spin filters with 10 kDa cut-off to about 30 µl before starting the protocol. Afterwards, samples were desalted using C18 Stop and Go Extraction (STAGE) [26] and dried by vacuum centrifugation.

### Liquid chromatography–tandem mass spectrometry – LC-MS/MS

Liquid chromatography-tandem mass spectrometry (LC-MS/MS) was used for label-free quantification (LFQ) of the proteolytic peptides. Two different setups were used. Mouse samples were analyzed on an Easy-nLC 1200 coupled to a Q-Exactive HF mass spectrometer (Thermo Scientific, Waltham, MA, US) as previously described for microglia [19] using data-independent acquisition and murine CSF samples [28] applying data dependent acquisition.

For human derived samples (hiMGL and hCSF), peptides were separated using a NanoElute nano HPLC equipped with either 30 cm (hCSF samples) or 15 cm (hiMGL samples) 75 μm ID columns packed with ReproSil-Pur 120 C18-AQ, 1.9 μm stationary phase (Dr. Mais GmbH, Germany) applying a 120 min gradient for lysates or a 70 min for hiMGL conditioned media and hCSF, which was online coupled to a TimsTOFpro mass spectrometer (Bruker, Germany). Samples were analyzed using DIA parallel accumulation serial fragmentation. For hCSF, one scan cycle induced one MS1 full scan followed by 2 rows of 50 sequential DIA windows with 18 m/z width for peptide fragment ion spectra with an overlap of 1 m/z covering a scan range of 350 to 1200 m/z (hCSF). The ramp time was fixed to 166 ms and 5 windows were scanned per ramp. This resulted in a total cycle time of 3 s. For hiMGL samples, one scan cycle included one MS1 full scan followed by 2 rows of 40 sequential DIA windows with 22 m/z width for peptide fragment ion spectra with an overlap of 1 m/z covering a scan range of 350 to 1200 m/z. The ramp time was fixed to 100 ms and 4 windows were scanned per ramp. This resulted in a total cycle time of 2.1 s.

### Mass spectrometry data analysis and label free quantification

Database search and label free quantification was performed using the MaxQuant software package (maxquant.org, Max-Planck Institute Munich) [29] for DDA and Spectronaut (Biognosys, CH) or DIA-NN (https://github.com/vdemichev/DiaNN, Version 1.8) [30]. The analysis of human CSF samples of the ALLFTD study was performed together with CSF samples of other origin than the ALLFTD, for which the data is not presented here. In total, 43 samples were analyzed using the DIA-NN (https://github.com/vdemichev/DiaNN, Version 1.8) [30], with which the normalization and library generation was performed. As we were specifically interested in FTD and the effect of GRN mutations on the human CSF proteome, we proceeded with the data only obtained from the ALLFTD study. The MS data were searched against canonical fasta databases including one protein per gene of Mus musculus and Homo sapiens from UniProt. For visualizations, unless stated otherwise, proteins are denoted with the gene name. For DIA of mouse microglia, a spectral library generated with samples of microglia with APPPS1 mice [19] was used for data analysis with Spectronaut. DIA-PASEF data from human CSF and iPSC derived human microglia were analyzed with DIA-NN using a library free search. Trypsin was defined as a protease with cleavage specificity for C-terminal of K and R. Acetylation of protein N-termini and oxidation of methionines were defined as variable modifications. Carbamidomethylation of cysteines was defined as fixed modification. For the database search, two missed cleavages were allowed. False discovery rate (FDR) for both proteins and peptides was adjusted to 1%.

### Statistical analysis

LFQ values were used for relative quantification, log2 transformed and statistically analyzed using Perseus (Version 1.6.15.0) [31] and GraphPad Prism (Version 9.3.1). Proteins were considered quantifiable when LFQ intensities could be detected in at least three biological replicates per experimental group. No data imputation was performed to replace missing LFQ values. Unless stated otherwise, an unpaired two-tailed Student’s *t*-test was applied to evaluate the significance of proteins with changed abundance. In addition, multiple hypothesis correction was performed using a permutation-based FDR estimation [32] (threshold: FDR = 0.05, s_0_ = 0.1) or the method of Benjamini, Krieger and Yekutieli [33] (threshold: FDR = 0.05). Of note, the FDR correction was not considered for the visualization of the data nor the selection of candidates, but is available in the supplementary data. For correlation analysis Spearman correlation was used (two-tail, 95% confidence interval), with the criteria that each selected protein was detected in at least three participants. For multiple comparison (comparing more than two groups), ordinary one-way ANOVA and Tukey’s test were used. Significance was indicated accordingly: “*” = p < 0.05, “**” = p < 0.01, “***” = p < 0.001; “n.s.” = non-significant. Diagnostic performance of each of the panel 6 proteins, as well as for panel 6 as a whole, was assessed using receiver-operating characteristic (ROC) curve analysis and area under the curve (AUC) scores were calculated with 95% confidence using DeLong’s test.

## Results

### Microglial activity state dependent proteomic signatures in mice correlate with transcriptomic signatures and are partially reflected by the CSF proteome

**Fig. 1:**
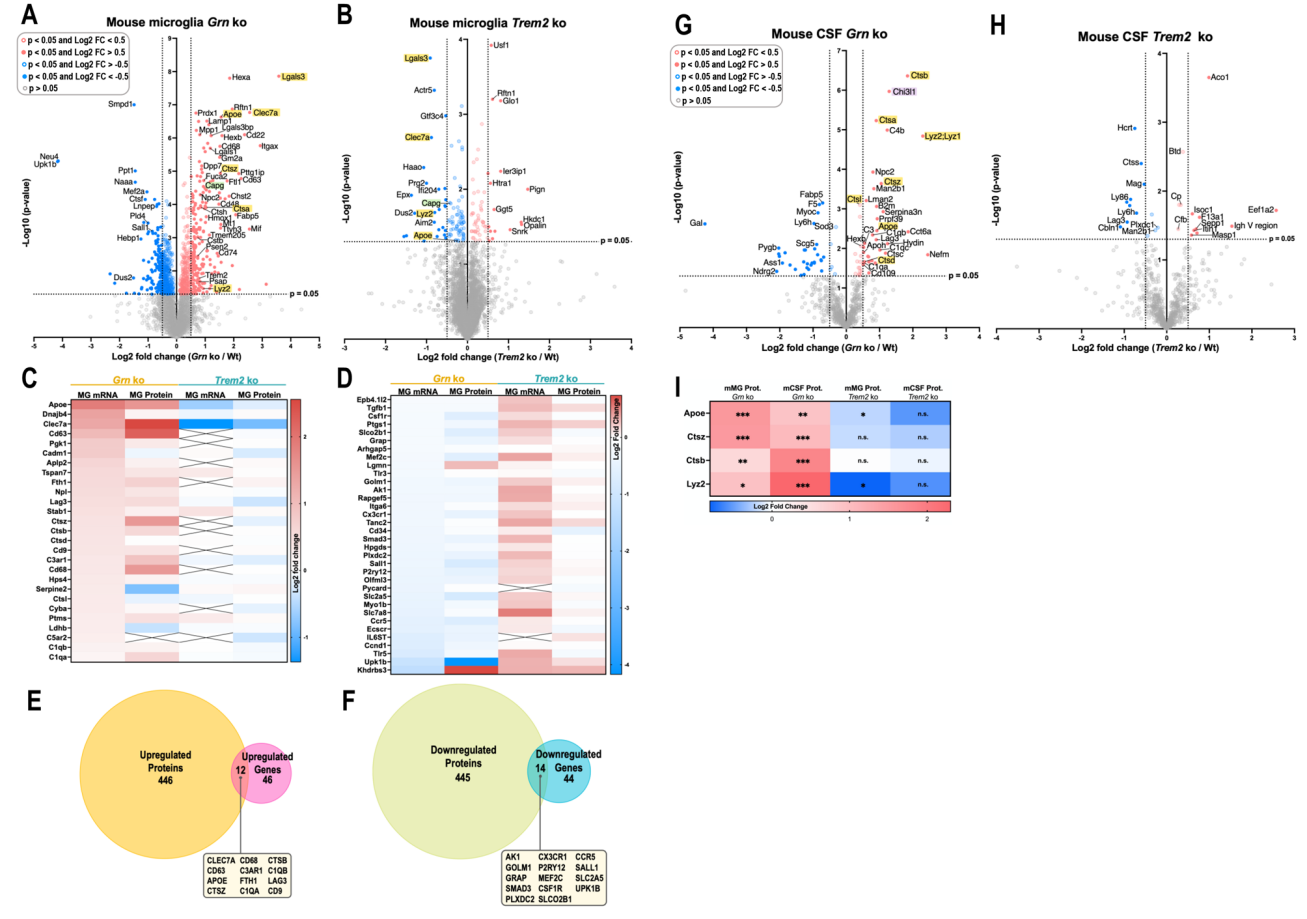
Microglial activation markers in *Grn* ko and *Trem2* ko are consistently changed on mRNA and protein level in mice and partially reflected in the mouse CSF proteome. **(A-B)** Volcano plots showing upregulated (red) and downregulated (blue) proteins in microglia isolated from mice with the following genotypic comparisons: **(A)***Grn* ko / Wt and **(B)***Trem2* ko / Wt. Selected cut-off values: p-value < 0.05 (colored dots) and − 0.5 > Log2 FC > 0.50 (filled dots). The -log10 transformed p-value of each protein is plotted against its log2 fold change. **(C-D)** Log2 fold-changes (relative to age-matched Wt) of microglial expressed mRNAs and related proteins, show very similar transcriptomic and proteomic changes. mRNA signatures were generated in independent samples from Cd11b-positive microglia isolated from 5.5 months old *Grn* ko and *Trem2* ko mice, respectively [11]. Crosses indicate missing values. **(E-F)** Comparing microglial proteome and transcriptome of *Grn* ko mice. **(E)** Significantly upregulated proteins (orange) versus significantly upregulated genes (pink). Overlap of 12 markers significantly upregulated on both protein and mRNA level (text box). **(F)** Significantly downregulated proteins (green) versus significantly downregulated genes (blue). Overlap of 14 markers significantly downregulated on both protein and mRNA level (text box). Selected cut-off values: p-value < 0.05. **(G-H)** Comparing upregulated (red) and downregulated (blue) proteins in mouse CSF with the following genotypic comparisons: **(G)***Grn* ko / Wt and **(H)***Trem2* ko / Wt. Cathepsins, Lyz2, as well as Apoe and Lgals3 are marked in yellow, while Chi3l1 (a biomarker for neuroinflammation) is marked in purple. Other proteins of interest are marked in green. Selected cut-off values: p-value < 0.05 and − 0.5 > Log2 FC > 0.50. **(I)** Changes (relative to age-matched Wt) of Apoe, Ctsz, Ctsb, and Lyz2 are consistent in the proteome of isolated microglia and in the proteome of CSF sampled from the same mice. Statistical differences were calculated using Student’s t-test. * = p-value < 0.05, ** = p-value < 0.01, *** = p-value < 0.001; n.s. = non-significant. False discovery rate (FDR) was not considered for the presented visualizations. All presented proteomic data was obtained by LC-MS/MS. Proteomic data were generated from 12 months old mice using the following experimental groups: n (*Grn* ko microglia) = 4, n (*Trem2* ko microglia) = 8, n (Wt microglia) = 7, n (*Grn* ko CSF) = 4, n (*Trem2* ko CSF) = 4, n (Wt CSF) = 6

We used a mass spectrometry-based approach to compare the proteome of microglia isolated from 12-months-old *Grn* ko and *Trem2* ko mice and their corresponding age-matched control (Wt) mice (Fig. 1A-B). Microglia from 12 months old *Grn* ko mice showed an upregulation of markers associated with microglia activation, such as Apoe, Lgals3, Lyz2, and Clec7a. In contrast, these activation-associated markers were downregulated in the age-matched *Trem2* ko mice (Fig. 1A-B). The proteome changes of the isolated microglia matched very well the transcriptomic signatures of the *Grn* ko or *Trem2* ko mice (Fig. 1C-D) [11]. This is seen with the microglial activation signature in the *Grn* ko mice, which showed a significant upregulation of Apoe, Clec7a, Cd63, Ctsz, Cd68, Fth1, Ctsb, and Cd9 proteins [11, 12, 34–39] in both proteomic and transcriptomic datasets (Fig. 1E). Similarly, markers indicating a homeostatic microglial signature, such as P2ry12, Cx3cr1, and Sall1 were significantly downregulated on both mRNA and protein level in the *Grn* ko mice (Fig. 1F).

With the microglial mRNA signature confirmed at the proteome level, we next investigated the proteome of mouse CSF collected pre-mortem from the same mice used for the isolation of microglia. The CSF proteome of the *Grn* ko mice showed several significant changes compared to age-matched Wt. Among the most significantly changed proteins within the CSF proteome of the *Grn* ko, we identified increased levels of Ctsb, Ctsd, Apoe, and Lyz1/2 similar to the microglia proteome (Fig. 1G), which is again consistent with their increase at the mRNA level in the DAM signature [36, 39]. In contrast, the homeostatic signature observed in the *Trem2* ko mouse microglia did not show pronounced changes in the CSF proteome compared to age-matched Wt mice (Fig. 1H). In general, the changes observed in the CSF proteome of mice are more subtle than those observed in the proteome of isolated mouse microglia (Fig. 1A-B). Nevertheless, DAM-like changes were detected in the CSF and microglial proteome of *Grn* ko mice as indicated by the consistent increase of the DAM markers Apoe, Ctsb, Ctsz, and Lyz2 (Fig. 1I) [19, 36, 39]. Noteworthy, besides the DAM-like changes, Chi3l1 (also known as YKL-40), a biomarker for neuroinflammation, showed a significant increase in the CSF of *Grn* ko mice, which is in line with elevated levels observed in the CSF of humans with neurodegenerative disorders (Fig. 1G) [40, 41].

### Proteomic signatures of *Grn* ko mouse microglia correlate with human iPSC derived *GRN* ko microglia

**Fig. 2:**
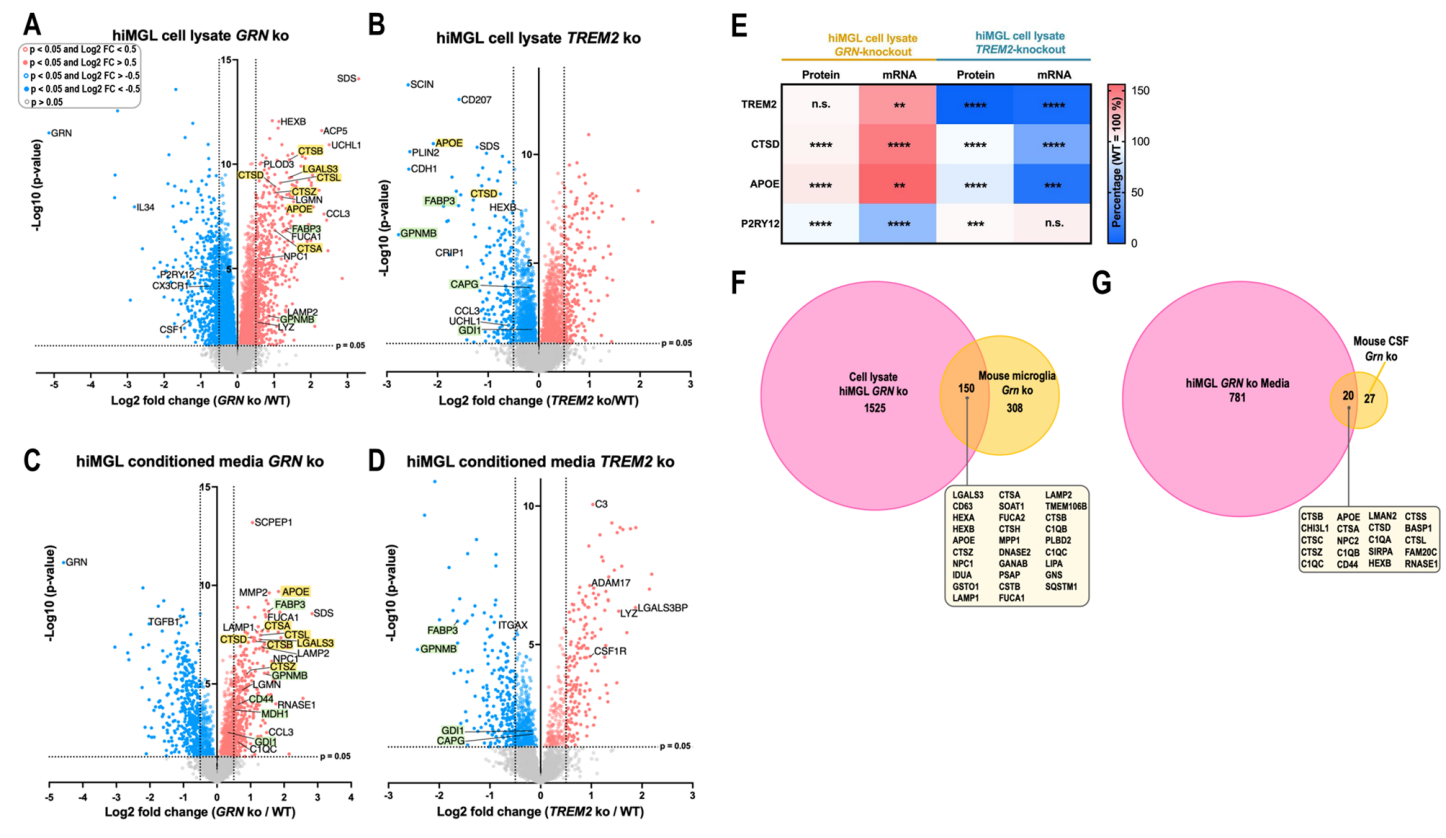
The protein signature of activated mouse microglia is comparable with hiMGL and associates well between mouse CSF and the hiMGL secretome. **(A-B)** Comparing upregulated proteins (red) versus downregulated proteins (blue) with significant changes indicated as colored dots, in the proteome of iPSC-derived microglia (hiMGL) with the following genotypic comparisons: **(A)** *GRN* ko / WT and **(B)** *TREM2* ko / WT. (**C-D)** Comparing proteomic changes in conditioned media from hiMGL (secretome) with the following genotypic comparisons: **(C)** *GRN* ko / WT **(D)** *TREM2* ko / WT. Cathepsins, as well as APOE and LGALS3 are marked in yellow. Other proteins of interest are marked in green. Selected cut-off values: p-value < 0.05 (colored dots) and − 0.5 > Log2 FC > 0.50 (filled dots). The -log10 transformed p-value of each protein is plotted against its log2 fold change. **(E)** Marker proteins related to microglia activation showed significant consistency between the proteome (first and third column) and transcriptome (second and fourth column) in hiMGL cell lysates. mRNA data were obtained by qPCR. **(F)** Proteins significantly (p-value < 0.05) upregulated in lysates of hiMGL *GRN* ko (pink) and *Grn* ko mice (orange). 150 proteins were significantly upregulated in both models (a selection of overlapping proteins is shown in the box). **(G)** Proteins significantly (p-value < 0.05) upregulated in the conditioned media of *GRN* ko hiMGL (pink) and CSF sampled from 12 months old *Grn* ko mice (orange). 20 proteins were significantly upregulated in both mouse CSF and conditioned media of hiMGL (box). Statistical differences were calculated using Student’s t-test. * = p-value < 0.05, ** = p-value < 0.01, *** = p-value < 0.001; n.s. = non-significant. False discovery rate (FDR) was not considered for the presented visualizations. All presented proteomic data were obtained by LC-MS/MS. Proteomic data were generated from hiMGL using the following experimental groups: n (*GRN* ko cell lysate) = 6, n (*TREM2* ko cell lysate) = 6, n (WT cell lysate) = 6, n (*GRN* ko conditioned media) = 6, n (*TREM2* ko conditioned media) = 6; n (WT conditioned media) = 6

To examine the potential translational applicability of our proteomic results from mouse microglia and CSF, we investigated proteomic changes in cell lysates and conditioned media of hiMGL harboring a *GRN* ko or *TREM2* ko (Appendix Figure S2) [20]. In line with the results obtained with mouse microglia, a larger number of proteins were significantly upregulated in the *GRN* ko hiMGL compared to *TREM2* ko hiMGL (Fig. 2A-B). Cathepsins (CTS) including CTSA and CTSZ, but also APOE and LGALS3 were significantly upregulated in the *GRN* ko hiMGL compared to WT control, which is in line with our findings in the microglia proteome of *Grn* ko mouse (Fig. 1 A).

Next, we analyzed the hiMGL secretome (conditioned media) (Fig. 2C-D). The proteomic signature observed in lysates of *GRN* ko hiMGL was reflected in conditioned media of *GRN* ko hiMGL. For example, several cathepsins (CTSA, CTSB, CTSD, CTSL, and CTSZ) were significantly increased in lysates and media of *GRN* ko hiMGL (Fig. 2A and C). Furthermore, the hiMGL models showed a robust correlation between the microglial transcriptome and proteome. For example, proteins with strong association to microglial activation, such as TREM2, APOE and CTSD were all upregulated on both, mRNA and protein level in the *GRN* ko hiMGL, compared to WT (Fig. 2E and Appendix Figure S2A). Consistent with the activation state of *GRN* ko hiMGL, levels of the homeostatic marker P2RY12 were significantly reduced in the *GRN* ko hiMGL transcriptome as well as the proteome (Fig. 2E and Appendix Figure S2A). In contrast, P2RY12 protein levels are slightly increased in the *TREM2* ko hiMGL, which are locked in a homeostatic state (Fig. 2E and Appendix Figure S2A) [11, 12, 20]. The microglial proteome signatures observed in mice were comparable with the changes measured in hiMGL. In total, 33% of proteins significantly upregulated in *Grn* ko mouse microglia were also significantly upregulated in the cell lysate of *GRN* ko hiMGL (Fig. 2F). In the secretome, > 42% of proteins significantly upregulated in CSF of *Grn* ko mice were also significantly upregulated in the secretome of *GRN* ko hiMGL (Fig. 2G). This suggests that the proteomic signature for microglial activation that we capture with our mouse models are at least in part conserved between mouse and human.

As in our mouse models, the changes in the *TREM2* ko hiMGL model are more subtle than those in *GRN* ko hiMGL. Comparison of the number of significantly upregulated proteins in cell lysates of *GRN* ko hiMGL (1675 proteins (Fig. 2F)) versus significantly upregulated proteins in lysates of *TREM2* ko hiMGL (1247 proteins (Appendix Figure S2B), reveals that the *GRN* ko hiMGL have 34% more proteins with significantly increased abundance compared to the *TREM2* ko hiMGL. With a lower number of significantly upregulated proteins in the *TREM2* ko models, the overlaps are also limited when comparing the microglia proteome of *Trem2* ko mice with *TREM2* ko hiMGL, resulting in an overlap of only 5 proteins (GLO1, PRPS2, HPRT1, SORT1, and DHX36) (Appendix Figure S2B). Of note, no proteins overlapped when comparing the 13 significantly increased proteins in the *Trem2* ko mouse CSF versus the 316 proteins with higher abundance in the secretome of *TREM2* ko hiMGL (Appendix Figure S2C).

### Consistent changes in the CSF proteome of symptomatic *GRN* mutation carriers and *GRN* ko hiMGL

**Fig. 3:**
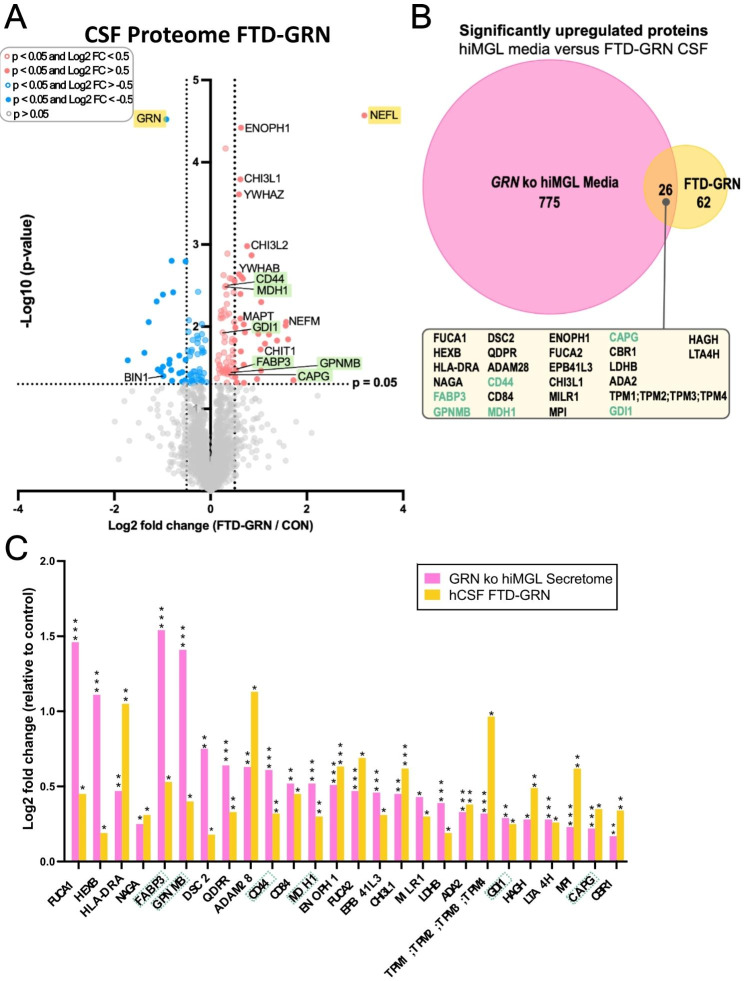
Overlapping proteomic changes observed in the CSF proteome of symptomatic GRN carriers and the secretome of GRN ko hiMGL. (**A)** Comparison of the CSF proteome of symptomatic heterozygous *GRN* mutation-carriers (FTD-GRN, n = 11) and healthy controls (CON, n = 12). Significantly upregulated proteins (red) versus significantly downregulated proteins (blue) are shown. Selected cut-off values: p-value < 0.05 (colored dots) and − 0.5 > Log2 FC > 0.50 (filled dots). The -log10 transformed p-value of each protein is plotted against its log2 fold change. (**B)** Identification of microglial activity state-dependent proteins by comparing the significantly upregulated proteins in human CSF of FTD-GRN patients (orange) to the significantly upregulated proteins detected in the conditioned media of *GRN* ko hiMGL (pink). In the CSF of FTD-GRN patients, 88 proteins were significantly upregulated compared to healthy non-carriers. 26 (30%) of these proteins were also detected as significantly upregulated in the conditioned media of hiMGL lacking *GRN* compared to media of WT hiMGL (box). **(C)** Quantitative comparison of the 26 proteins detected as significantly upregulated in both, the human CSF (hCSF) of FTD-GRN patients (orange) and the media of *GRN* ko hiMGL (pink) presented as Log2 Fold Change in relation to respective control (CON and WT, respectively). Proteins of interest are marked in yellow and green. Statistical differences were calculated using Student’s t-test. * = p-value < 0.05, ** = p-value < 0.01, *** = p-value < 0.001, n.s. = non-significant. False discovery rate (FDR) was not considered for the presented visualizations. All presented proteomic data were obtained by LC-MS/MS.

For further validation of our proteomic results from mice and hiMGL, we analyzed the CSF proteome of human *GRN* mutation carriers. In total, the cohort included 11 symptomatic heterozygous *GRN* mutation carriers (FTD-GRN; MC = mutation carrier), of which 3 patients were diagnosed with mild cognitive impairment (MCI) at the timepoint of CSF sampling. In addition, the CSF of 12 non-symptomatic controls (CON; NC = non-carrier) without *GRN* mutations were included (Appendix Table S1). As expected, GRN was significantly less abundant in the CSF of FTD-GRN patients compared to healthy controls (Fig. 3A). On the other hand, levels of neurofilament light (NEFL, also commonly abbreviated as Nfl) were significantly increased in the CSF of FTD-GRN patients, indicating massive neurodegeneration (Fig. 3A). Comparison of the CSF proteome of FTD-GRN patients to the proteome data obtained from conditioned media of *GRN* ko hiMGL secretome provided the identification of 26 proteins with increased abundance in both fluids (p-value < 0.05, not considering FDR-correction) (Fig. 3B-C). When comparing these 26 proteins with our proteomic data derived from the *Grn* ko mouse microglia, a statistically significant upregulation is observed for four proteins, which therefore show a consistent increase across species and models: FUCA1, HEXB, FUCA2, and CAPG. In the CSF of *Grn* ko mice, the increased levels of three proteins found in the human CSF also reached a statistical significance: HEXB, CD44, and CHI3L1 (Appendix Figure S3).

### Identification of six microglia activation-dependent proteins

**Fig. 4:**
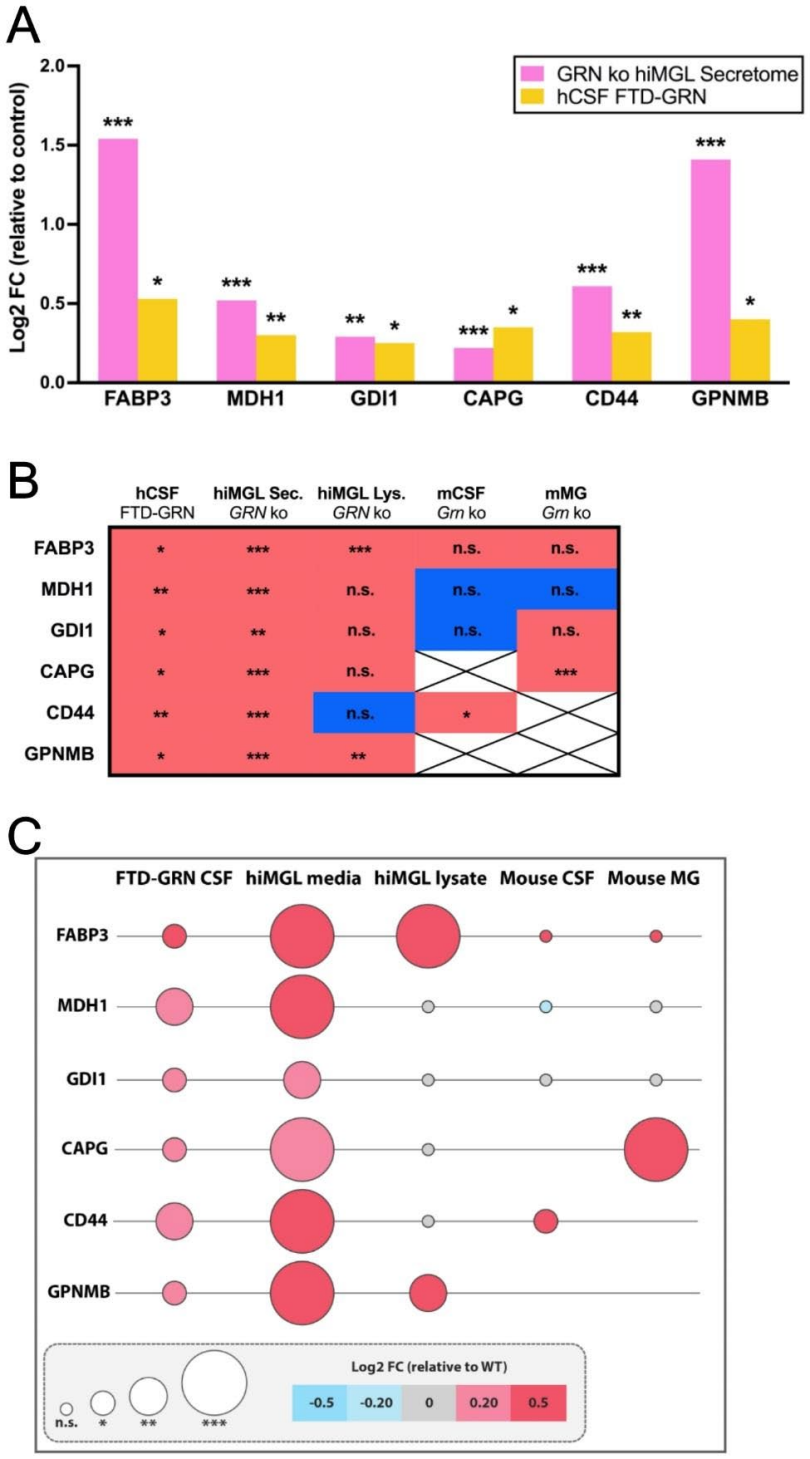
Identification of six microglial activation-dependent proteins. **(A)** Log2 transformed abundances of panel 6 proteins (FABP3, MDH1, GDI1, CAPG, CD44, and GPNMB) in the secretome of *GRN* ko hiMGL (pink) and in CSF of symptomatic FTD-GRN patients (orange). The abundance of each group is normalized to the abundances measured in control groups, secretome of WT hiMGL and CSF levels of healthy non-carrier (CON). **(B)** Binary representation of the up- (red) and downregulation (blue) of each protein in hCSF of FTD-GRN patients, conditioned media of *GRN* ko hiMGL, cell lysates of *GRN* ko hiMGL, CSF (mCSF) of *Grn* ko mice, and microglia (mMG) of *Grn* ko mice. Crosses indicate missing values. **(C)** Comparison of Log2 transformed abundance of panel 6 proteins in CSF from FTD-GRN patients, conditioned media from *GRN* ko hiMGL, *GRN* ko hiMGL cell lysates, CSF and microglia from *Grn* ko mice. Circle size indicates statistical significance based on p-value obtained from Student’s t-test, * = p-value < 0.05, ** = p-value < 0.01, *** = p-value < 0.001, n.s. = non-significant. False discovery rate (FDR) was not considered for the presented visualizations. Color intensity indicates the degree of up- (red) or downregulation (blue). Unchanged abundances are indicated in gray and are defined by -0.2 < Log2 fold change < 0.2

By comparing the 26 proteins that were upregulated in both the secretome of *GRN* ko hiMGL and in the CSF of FTD-GRN patients (Fig. 3B and Appendix Table S2) we identified six proteins, which we refer to as panel 6, including fatty acid binding protein 3 (FABP3), malate dehydrogenase 1 (MDH1), GDP dissociation inhibitor-1 (GDI1), macrophage-capping protein (CAPG), CD44, and glycoprotein NMB (GPNMB) (Figs. 3A–C and 4A). All proteins of panel 6 were significantly increased in the CSF of FTD-GRN patients and in the conditioned media of *GRN* ko hiMGL. FABP3 and GPNMB are the only proteins from panel 6 that were also significantly upregulated in the cell lysate of the *GRN* ko hiMGL. In *Grn* ko mice, CD44 was the only protein within panel 6 that was significantly upregulated in CSF, while CAPG was the only protein significantly upregulated in mouse microglia (Fig. 4B-C and Appendix Figure S3). Investigating each of the six proteins separately, within the *GRN* ko hiMGL secretome and the CSF of FTD-GRN patients, FABP3 stands out with increasing CSF levels of > 50% in FTD-GRN patients compared to healthy controls (Appendix Figure S4A). Noteworthy, the FABP3, MDH1 and GDI1 CSF levels in the three *GRN* mutation carriers with less prominent symptoms and diagnosed with MCI, are below average of the FTD-GRN group (Appendix Figure S4A-C). CSF levels of the remaining three proteins (CAPG, CD44, and GPNMB) were significantly elevated in *GRN* mutation carriers, but without any reducing effect caused by the MCI diagnosis (Appendix Figure S4D-F).

### Panel 6 proteins distinguish FTD-GRN patients from controls and correlate with levels of CHI3L1

**Fig. 5:**
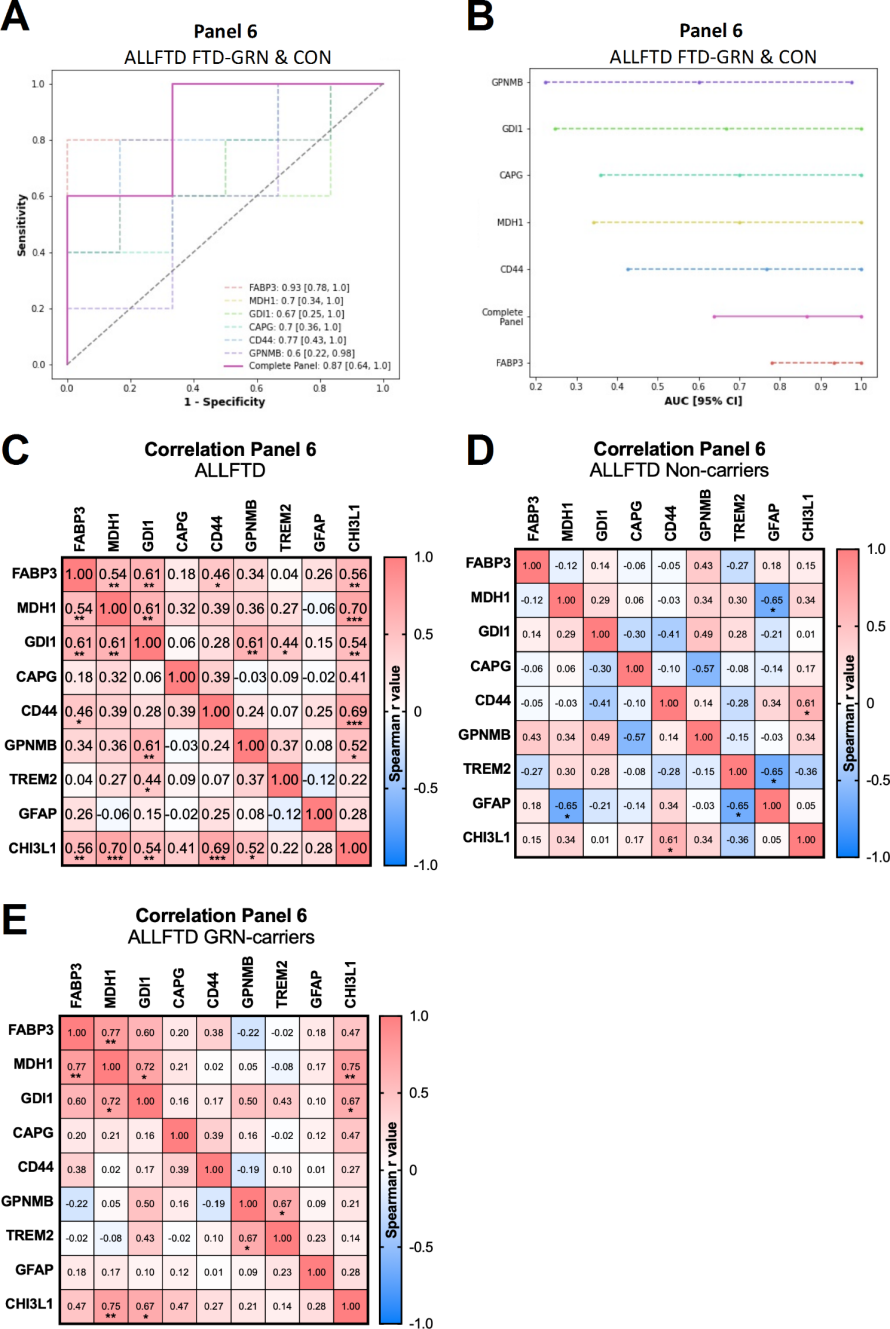
Panel 6 proteins successfully distinguishing FTD-GRN patients from controls and correlate with levels of CHI3L1. **(A-B)** ROC curve analysis indicating the diagnostic performance of panel 6 (AUC = 0.87, 95% CI = 0.64 to 1.0) to separate cohort participants as affected (FTD-GRN) versus not affected (CON). **(C-E)** Correlation analysis of CSF levels of panel 6 proteins and TREM2 (microgliosis), GFAP (astrogliosis), and CHI3L1 (also known as YKL-40, neuroinflammation) within **(C)** the entire ALLFTD cohort (n (ALLFTD) = 23)), within **(D)** the control group only (n (NC) = 12), and within **(E)** the symptomatic FTD-GRN group (n (FTD-GRN) = 11). CSF levels of TREM2 (microgliosis), GFAP (astrogliosis), and CHI3L1 (also known as YKL-40, neuroinflammation) are included based on the quantitative data obtained from LC-MS/MS analysis. Each cell is colored and labeled according to Spearman r value; Spearman r > 0.5 is normally considered to indicate a strong correlation, while Spearman r > 0.75 is considered to indicate a very strong correlation.* = p-value < 0.05, ** = p-value < 0.01, *** = p-value < 0.001, n.s. = non-significant. FDR was not considered for the presented visualizations

The diagnostic power of panel 6, to distinguish FTD-GRN patients from healthy controls was evaluated using ROC curve analysis. When combined, the panel 6 proteins generated an AUC of 0.87 (95% CI = 0.64 to 1.0) (Fig. 5A). Notably, our results revealed that FABP3 alone generated an AUC of 0.93 (95% CI = 0.78 to 1.0) (Fig. 5A-B).

For further characterization of panel 6, the correlation between each of the six proteins was investigated within the entire cohort (Fig. 5C) as well as within each group (healthy non-carriers and FTD-GRN) (Fig. 5D-E). Although not significantly altered in the CSF data, we included known markers for microgliosis (TREM2), astrogliosis (GFAP), and neuroinflammation (CHI3L1, also known as YKL-40) for correlation analysis. A significant correlation was observed between FABP3, MDH1, and GDI1 (Fig. 5C). The levels of these proteins also significantly correlated with the levels of CHI3L1 (Fig. 5C). In general, the observed correlations were weaker in the non-carriers compared to the *GRN* mutation carriers (Fig. 5D-E). Strong correlations (defined by Spearman r > 0.5) were observed between GPNMB-CHI3L1, GDI1-GPNMB, GDI1-CHI3L1, FABP3-MDH1, FABP3-CHI3L1, MDH1-CHI3L1, CD44-CHI3L1, GDI1-MDH1, and FABP3-GDI1 (Fig. 5C). Very strong correlations (defined by Spearman r > 0.75) were observed between FABP3-MDH1 and MDH1-CHI3L1 in the FTD-GRN group (Fig. 5E).

### CSF levels of FABP3, MDH1, and GDI1 are significantly elevated in AD and mild cognitive impairment and driven by Aβ

**Fig. 6:**
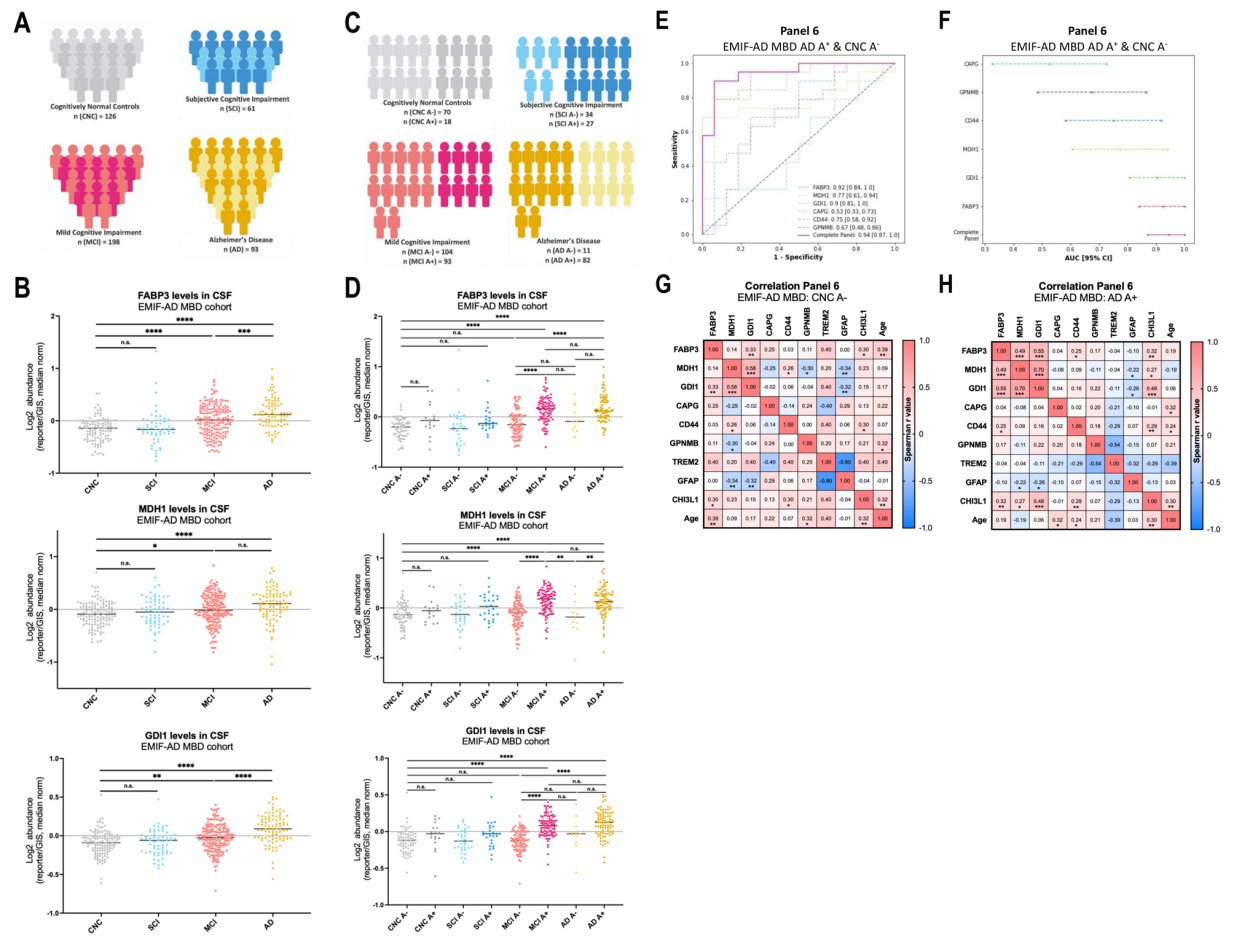
CSF levels of FABP3, MDH1, and GDI1 correlates with each other and are significantly elevated in an amyloid dependent manner in MCI and AD. (**A)** Overview of the EMIF-AD MBD cohort with stratification according to diagnosis only. **(B)** CSF levels of FABP3 (top), MDH1 (middle), and GDI1 (bottom) in cognitively normal controls (CNC) (gray, n = 126), SCI (blue, n = 61), MCI (pink, n = 198), and AD (yellow, n = 93). CSF levels are defined by Log2 abundance, normalized to the abundance measured in the TMT-reference control channel [15] and the median of each individual. **(C)** Overview of the EMIF-AD MBD cohort with stratification according to diagnosis and amyloid status as assessed by the levels of CSF amyloid-β_1−42_[22]. **(D)** CSF levels of FABP3 (top), MDH1 (middle), and GDI1 (bottom) in CNC A^−^ (light gray, n = 70), CNC A^+^ (dark gray, n = 18), SCI A^−^ (light blue, n = 34), SCI A^+^ (dark blue, n = 27), MCI A^−^ (light pink, n = 104), MCI A^+^ (dark pink, n = 93), AD A^−^ (light yellow, n = 11), and AD A^+^ (dark yellow, n = 82). A^−^ = amyloid normal, A^+^ = amyloid abnormal [15]. Ordinary one-way ANOVA (alpha = 0.05). **(E-F)** ROC curve analysis indicating the diagnostic performance of panel 6 (AUC = 0.94, 95% CI = 0.87 to 1.0) to separate cohort participants as affected (amyloid-positive AD) versus not affected (amyloid-negative CNC). (**G-H)** Correlation analysis of panel 6 proteins and TREM2 (microgliosis), GFAP (astrogliosis), and CHI3L1 (also known as YKL-40, neuroinflammation) in **(G)** amyloid-negative CNC and in **(H)** amyloid-positive AD patients. CSF levels are defined by Log2 abundance, normalized to the abundance measured in the TMT-reference control channel and the median of each individual. Each cell is colored and labeled according to Spearman r value; Spearman r > 0.5 is normally considered to indicate a strong correlation, while Spearman r > 0.75 is considered to indicate a very strong correlation. * = p-value < 0.05, ** = p-value < 0.01, *** = p-value < 0.001, n.s. = non-significant. FDR was not considered for the presented visualizations

For further validation, we investigated the abundances of the six selected proteins in a previously published proteomic dataset from the CSF of 478 participants of the EMIF-AD MBD cohort [15]. Within this cohort three of our six selected proteins were significantly altered between cohort groups stratified according to diagnosis, namely FABP3, MDH1, and GDI1 (Fig. 6A-B). The CSF levels of CAPG, CD44, and GPNMB did not differ between the cohort groups (Appendix Figure S5A-B). Of note, the CSF levels of FABP3, MDH1, and GDI1 were not only significantly elevated in participants diagnosed with AD, but also in the non-dementia classed MCI group when compared to CNC (Fig. 6B). Stratification according to amyloid status allowed further separation within the MCI and AD group. CSF levels of FABP3, MDH1, and GDI1 were significantly different within the MCI group, with a highly significant elevation in MCI cases with amyloid abnormalities compared to amyloid-negative MCI cases (Fig. 6C-D). Furthermore, CSF levels of MDH1 became significantly different within the AD group, when stratifying this group according to amyloid status (Fig. 6D).

The diagnostic power of panel 6, to distinguish amyloid-positive AD patients from amyloid-negative healthy controls was evaluated using ROC curve analysis. When combined, the panel 6 proteins generated an impressive AUC value of 0.94 (95% CI = 0.87 to 1.0) (Fig. 6E). Interestingly, our results revealed that FABP3, GDI1, and MDH1 are the best performing proteins among the six panel proteins, with AUC values of 0.92 (95% CI = 0.84 to 1.0), 0.90 (95% CI = 0.81 to 1.0), and 0.77 (95% CI = 0.61 to 0.94), respectively (Fig. 6E-F).

Finally, we found a similar correlation between FABP3, MDH1 and GDI1, as observed in the ALLFTD cohort (Figs. 5C and 6G-H). The observed correlation was stronger in the group of amyloid-positive AD patients compared to the amyloid-negative control group (CNC) (Fig. 6G-H). Further emphasizing the relevance of these three markers in a neuroinflammatory setting, all three markers showed a significant correlation with neuroinflammatory marker CHI3L1 within the amyloid-positive AD group (Fig. 6H).

## Discussion

Discrimination of microglial activity states is currently largely based on transcriptomic profiling [11, 34, 36, 39, 42–45]. We previously reported opposite microglial phenotypes in mice lacking TREM2 or GRN. In the absence of TREM2 microglia were locked in a homeostatic state, whereas *Grn* ko microglia were hyperactivated [11, 12]. We now made use of these findings and compared the opposite microglia signatures upon loss of TREM2 or GRN function on the proteome level in human and mouse models to explore potential markers for microglia activation and confirmed the translational applicability of our findings in CSF of AD and FTD-GRN patients.

The main finding of our study is the identification of six microglia activation-dependent markers, referred to as panel 6 including FABP3, MDH1, GDI1, CAPG, CD44, and GPNMB. These proteins are detectable and quantifiable in the conditioned media of hiMGL and in human CSF. Furthermore, they are all significantly increased in the conditioned media of *GRN* ko hiMGL, as well as in the CSF of FTD-GRN patients (Fig. 3B). We decided not to refer to these changes as microglia-specific, as these proteins are expressed by other cell types as well. Nevertheless, due to the fact that the *GRN* ko hiMGL are grown in a monoculture with a confirmed activation signature and very little contamination from other brain cells, the significant upregulation of these proteins in the conditioned media of the *GRN* ko hiMGL strongly supports that the changes that we observed are indeed dependent on the microglial activation status. Furthermore, panel 6 proteins where also upregulated in FTD-GRN patients, where microglia are known to be hyperactivated [13, 14]. Hence, we refer to our panel of six proteins as microglia activation-dependent. Our findings were further confirmed in a completely independent dataset derived from the EMIF-AD MBD cohort [15]. CSF levels of FABP3, MDH1, and GDI1, were significantly elevated in MCI and AD patients of the EMIF-AD MDB cohort (Fig. 6A-B). Strikingly, CSF levels of these three candidates were significantly different between MCI patients with and without detectable amyloid abnormalities (Fig. 6D), thus the changes in their concentrations appear to be driven by early deposition of Aβ. The separation of MCI patients according to their amyloid status, without the use of traditional AD biomarkers, is highly unique. Importantly, this separation was statistically significant for each of the three proteins independently from each other, which highlights the potential of each of these candidates for stratification purposes. This is in line with our previous findings in the DIAN cohort, where we found increased sTREM2 up to 21 years before the estimated year of symptom onset following immediately the deposition of amyloid [7]. Together, these findings support the concept that microglia respond to the earliest amyloid related challenge even before profound cognitive changes are detected. Furthermore, our study revealed a significant correlation between CSF levels of FABP3, MDH1, and GDI1 in the FTD cohort (ALLFTD) and the AD cohort (EMIF-AD MBD) (Fig. 5C H). In the ALLFTD cohort, CSF levels of GPNMB showed a correlation with CSF TREM2, a correlation that is significant in FTD-GRN patients but not in healthy controls (Fig. 5D-E). In addition, FABP3, MDH1, GDI1, CD44, and GPNMB individually correlated with the neuroinflammatory marker CHI3L1 (also known as YKL-40) [40, 41, 46, 47], providing further evidence that the levels of these markers are indeed linked to a microglial response (i.e. a change in their activity state) (Figs. 5C and 6H).

Further supporting our findings, abnormal CSF levels of individual members of panel 6 proteins have been reported in various neurodegenerative diseases: (1) FABP3 in cases of Parkinson’s disease with dementia (PDD), Dementia with Lewy bodies (DLB) [48], AD [48–50] and even in healthy individuals with confirmed amyloid pathology [51], (2) MDH1 in sporadic (sCJD) and genetic Creutzfeldt-Jakob disease (gCJD) [52, 53] and in AD [50], (3) GDI1 in AD [50], (4) CAPG in sporadic and genetic amyotrophic lateral sclerosis (ALS) [54], (5) CD44 in AD [50], and (6) GPNMB in sporadic and genetic amyotrophic lateral sclerosis (ALS) [54].

Importantly, our data also revealed changes of the proteome that are not translatable across the mouse and human models, such as protein levels of MDH1, ENOPH1, CD84, and EPB41L3, proteins that are upregulated in the hiMGL model and in human CSF, but downregulated in mice (Appendix Figure S3). The translational commonalities and differences reported here may therefore also be of importance for selecting and understanding microglial responses in different models. Furthermore, according to previously published data, protein levels of FABP3 and CAPG may increase in an age-dependent manner in microglia of amyloid mouse models, while the protein levels of MDH1 appear to decrease in the very same mice [19]. In studies investigating the microglial transcriptome, *GPNMB* has been reported as part of a DAM signature in mouse models with both, amyloid and tau pathology [55]. The translational commonalities and differences between mouse and human should especially be considered for transgenic murine models, which may show alterations in the proteome depending on age and neuropathological burden.

Biomarkers are not only relevant for clinical applications, but their changes may inform us about important metabolic abnormalities during disease onset and progression. FABP3 belongs to a family of proteins involved in uptake and metabolism of long-chain-fatty acids [56, 57], while GPNMB appears to be involved in cell proliferation [58]. Both may be highly relevant, since many if not all TREM2 agonistic antibodies boost microglial proliferation and ATV:TREM2, a brain penetrant anti-human TREM2 agonist also ameliorates lipid dysmetabolism [3, 59]. Furthermore, levels of cellular FABP3 have been reported to increase in adipocytes and hepatocytes upon chronic exposure to high lipid concentrations [56]. Finally, FABP3, together with APOE, were recently proposed as biomarkers for lipid metabolism in AD patients [49]. It is therefore likely that the elevated FABP3 levels that we observe in microglia reflect a response to the lipid burden that microglia are facing in diseased conditions [60] specifically upon GRN deficiency [61] but also in other neurodegenerative diseases. MDH1 plays a central role in the malate-aspartate shuttle and the TCA cycle, which have been linked to LPS-induced microglial activation [62]. Finally, GPNMB has been reported as highly expressed in microglial populations that either cluster around Aβ plaques or that are located distal to plaques but appear as amoeboid and with high lipid content [63]. Of note, the soluble variant of GPNMB acts as a ligand for CD44, which is associated with anti-inflammatory responses as well as de novo lipogenesis [64–66]. As previously suggested, lipid-related processes and metabolic reprogramming are of central importance in the context of microglial activation [67–72]. Moreover, many of the protective functions of TREM2 have been linked to the sensing, binding, uptake, and metabolism of lipids, which are required for the microglial response [73–77]. Of note, the currently used technology for in vivo assessing microglial activation makes use of translocator protein-positron emission tomography (TSPO-PET). TSPO-PET is based on the expression of TSPO protein, which is expressed at the mitochondrial membrane by multiple cell types throughout the body [78]. TSPO is not microglia-specific, but appears to be microglia activation-dependent [11, 79–82]. Interestingly, the main function of TSPO is to transport cholesterol across the mitochondrial membrane, where it can be further metabolized [83]. Taken together, the microglial response is highly dependent on lipid-related processes, which further supports our idea that changes in lipid metabolism reflect microglia activation.

Due to the relative rarity of *GRN*-mutations associated with FTD (representing 5–20% of all FTD cases [84]), the rather small cohort size is a limitation of our study. In addition to the small cohort size, the obtained clinical data for the participants of the ALLFTD is very limited, which may affect the statistical analysis and compromise the interpretation of the ROC analysis. For these reasons, we employ this model as a companion to conventional tests to provide further evidence of the protein panel’s involvement, but not as exclusive evidence of the diagnostic capacities of this panel. Within the ALLFTD cohort and EMIF-AD MBD cohort Caucasian participants are overrepresented, which limits our study in the sense that none of these cohorts represent the global population. Therefore, additional human studies are required to confirm the selected candidates.

### Electronic supplementary material

Below is the link to the electronic supplementary material.

Supplementary Material 1

Supplementary Material 2

Supplementary Material 3

Supplementary Material 4

Supplementary Material 5

Supplementary Material 6

## Data Availability

The codes used for data sharing are available from IP, SAM, and SFL. The data obtained from the EMIF-AD MBD cohort are available from Weiner et al. 2022 but restriction apply to the availability of these data and are only available upon request addressed to the original authors.

## Abbreviations

A+: Amyloid positive, abnormal levels of CSF amyloid-β_1−42_
A-: Amyloid positive, normal levels of CSF amyloid-β_1−42_
AD: Alzheimer’s disease
ALS: Amyotrophic lateral sclerosis
APOE: Apolipoprotein E
ARTFL: Advancing research & treatment for frontotemporal lobar degeneration
BAF: B allele frequencies
BSA: Bovine serum albumin
CAPG: Macrophage-capping protein
Cas9: CRISPR-associated endonuclease Cas9
CFD: Cutting frequency determination
CLEC7A: C-type lectin domain family 7 member A
CNC: Cognitively normal controls
CON: Control group of the ALLFTD cohort, non-symptomatic non-carriers
CRISPR: Clustered regularly interspaced short palindromic repeats
CSF: Cerebrospinal fluid
CTSA: Cathepsin A
CTSB: Cathepsin B
CTSD: Cathepsin D
CTSL: Cathepsin L
CTSZ: Cathepsin Z
CX3CR1: CX3C chemokine receptor 1
DAM: Disease-associated microglia
DAP12: DNAX activation protein of 12 kDa
DAPI: 4’,6-diamidino-2-phenylindole
DDA: Data dependent acquisition
DIA: Data independent acquisition
EDTA: Ethylenediaminetetraacetic acid
ELISA: Enzyme-linked immunosorbent assay
EMIF-AD MBD: European Medical Information Framework Alzheimer’s Disease Multimodal Biomarker Discovery
FABP3: Fatty acid binding protein 3
FASP: Filter aided sample preparation
FTD: Frontotemoral dementia
GDI1: GDP dissociation inhibitor-1
GPNMB: Glycoprotein non-metastatic B
GRN: Progranulin encoding gene
GWAS: Genome-wide association studies
HBSS: Hank’s balanced salt solution
hCSF: Human CSF
HEPES: N-2-hydroxyethylpiperazine-N-2-ethane sulfonic acid
hiMGL: Human-iPSC-derived microglia
HPLC: High-performance liquid chromatography
ID: Inner diameter
iPSC: Induced pluripotent stem cell
IVC: Individually ventilated cages
kDa: Kilodaltons
ko: Knockout
LC-MS: Liquid chromatography–mass spectrometry
LC-MS/MS: Liquid chromatography tandem mass spectrometry
LEFFTDS: Longitudinal evaluation of familial frontotemporal dementia subject
LFQ: Label-free quantification
LOAD: Late-onset AD
LPS: Lipopolysaccharide
LYZ2: Lysozyme C-2 (mouse)
MACS: Magnetic-activated cell sorting
MCI: Mild cognitive impairment
mCSF: Mouse CSF
MDH1: Malate dehydrogenase 1
MMF: Medetomidine-midazolam-fentanyl
mRNA: Messenger RNA
NaCl: Sodium chloride
NCRAD: National centralized repository for Alzheimer’s Disease and related dementias
P2RY12: P2Y purinoceptor 12
PBS: Phosphate-buffered saline
qPCR: Quantitiative PCR
RFLP: Restriction fragment length polymorphism technique
SCI: Subjective cognitive impairment
sgRNA: Single guide RNA
SNP: Single-nucleotide polymorphism
SP3: Single-pot, solid-phase-enhanced sample preparation
STET: Sodium chloride-Tris-Cl-EDTA-Triton X-100
sTREM2: Soluble TREM2
TREM2: Triggering receptor expressed on myeloid cells 2
TSPO: Translocator protein
TSPO-PET: Translocator protein 18 kDa-positron emission tomography
VTN: Vitronectin
WT: Wild-type
