# Supplementary figures and images for: A microglial activity state biomarker panel differentiates FTD-granulin and Alzheimer’s disease patients from controls
Source: Mol Neurodegener. 2023 Sep 29;18:70. doi: 10.1186/s13024-023-00657-w (PMC10543321; doi:10.1186/s13024-023-00657-w)

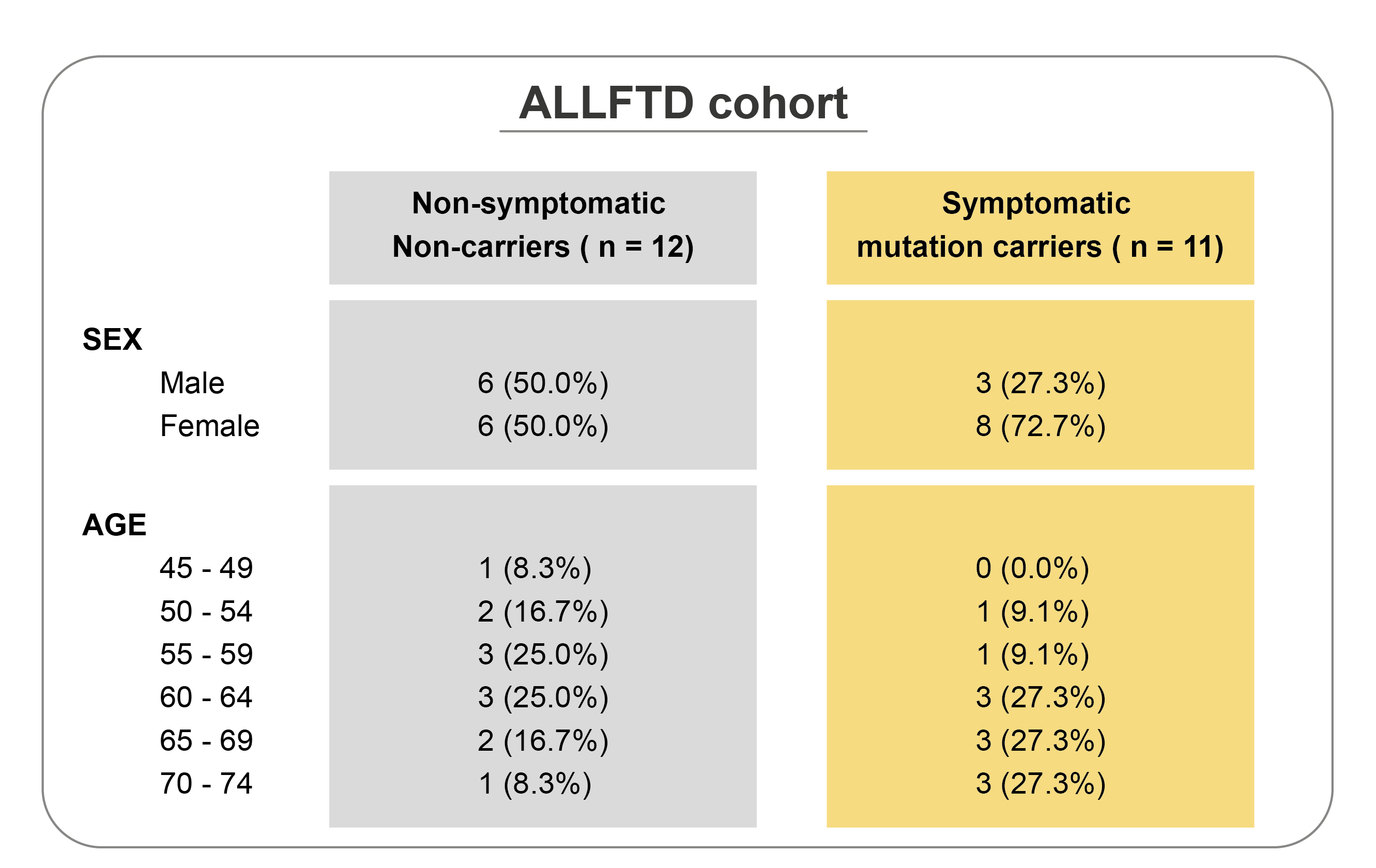


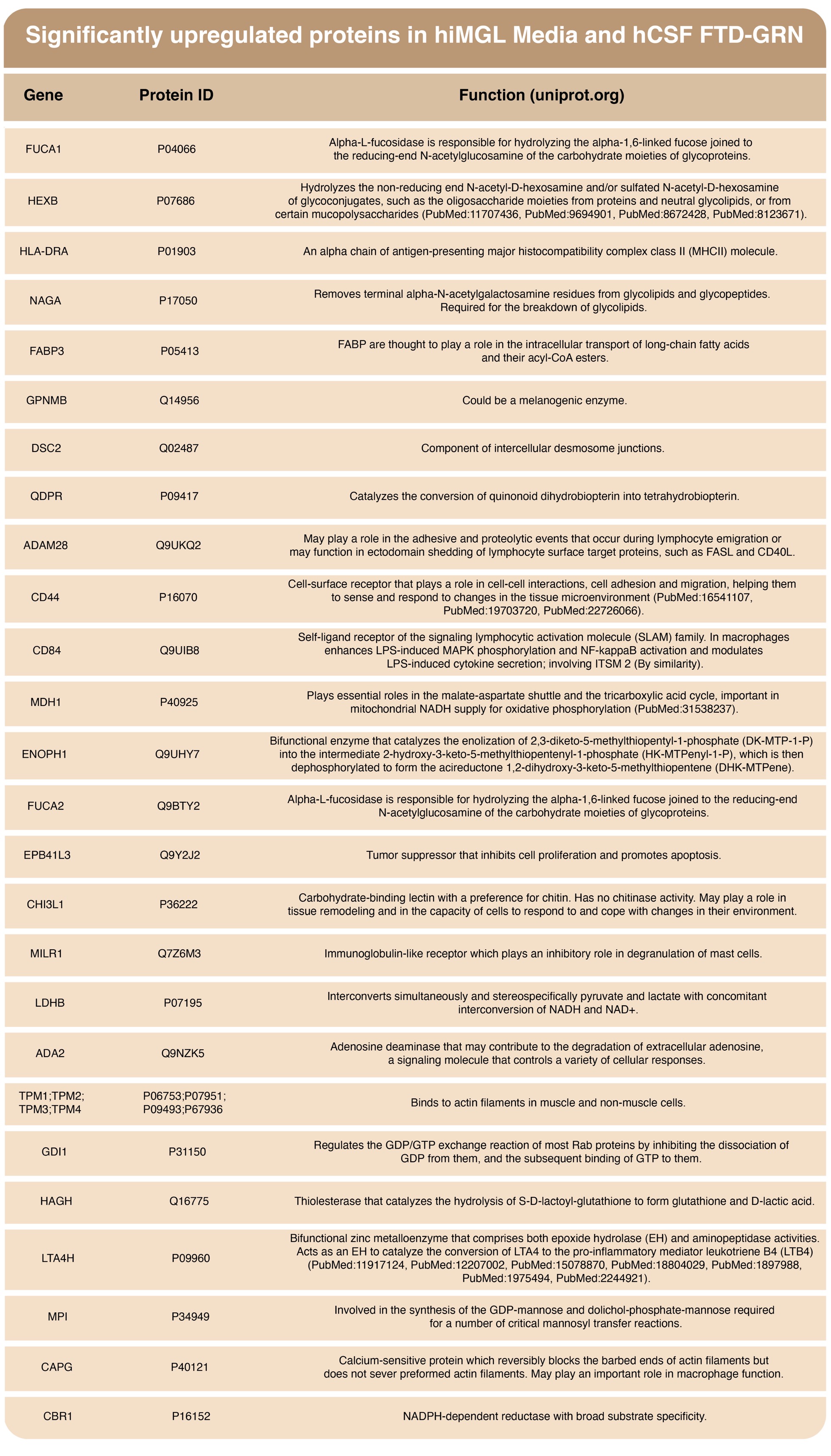

Supplement: Supplementary file 4 — Supplementary Material 4 [file 13024_2023_657_MOESM4_ESM.docx]

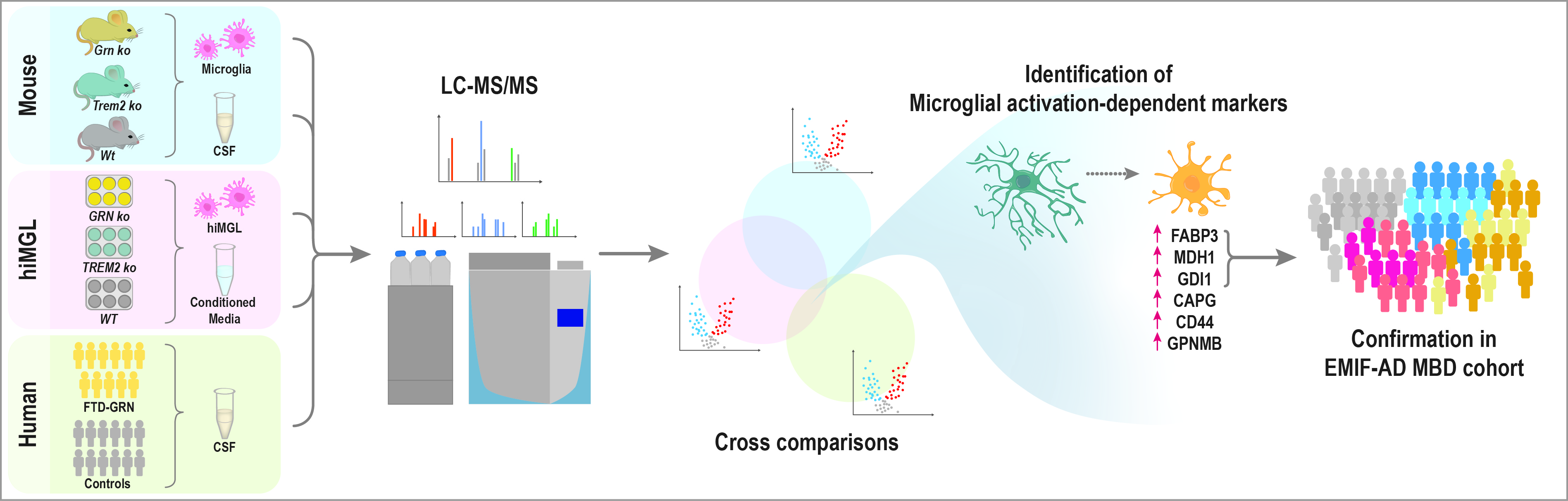

Supplement: Supplementary file 5 — Supplementary Material 5 [file 13024_2023_657_MOESM5_ESM.jpg]
