# Supplementary material for: A microglial activity state biomarker panel differentiates FTD-granulin and Alzheimer’s disease patients from controls
Source: Mol Neurodegener. 2023 Sep 29;18:70. doi: 10.1186/s13024-023-00657-w (PMC10543321; doi:10.1186/s13024-023-00657-w)
Supplement: Supplementary file 6 — Supplementary Material 6 [file 13024_2023_657_MOESM6_ESM.docx]

Dear Dr. Bu,

Therapeutic modulation of microglial activity is an innovative approach for treatment of Alzheimer’s disease (AD) and other neurodegenerative disorders and several TREM2 agonistic antibodies are already in clinical trials. However, we are missing biomarkers, which allow monitoring dynamic changes in microglial activities. Such biomarkers may also be very valuable for patient stratification and monitoring of microgliosis in clinical trials using anti-amyloid antibodies such as Lecanemab and Donanemab. To identify microglial activity state-dependent fluid phase biomarkers we made use of the two most opposite microglial activity states, which we have identified earlier (Götzl et al., EMBO Mol Med, 2019). In the Progranulin (*GRN*) knockout, microglia are hyperactivated, whereas in the *TREM2* knockout microglia are locked in a homeostatic state. Using explorative non-targeted proteomics, we searched for a panel of proteins, which in biological fluids may indicate activity changes of microglia. Proteomes of isolated microglia from the two knockout mouse models, as well as their cerebrospinal fluids were compared to proteomes from human iPSC derived *GRN*- and *TREM2* knockout microglia (cell lysates and conditioned media). For complete translation, relevant to human patients, we also analyzed the CSF proteome of FTD-GRN carriers. By comparing the most differentially changed proteins, we found a panel of six proteins, which allowed the separation of FTD-GRN patients from healthy controls. We then made use of already published data from a cross-sectional observational study (Weiner et al., Clin Proteomics, 2022) to investigate if our top hits are also changed during AD. Strikingly, three of these proteins were not only significantly increased in the CSF of symptomatic AD patients, but even in individuals with mild cognitive impairment (MCI). Interestingly, this observation appeared to be driven by amyloid pathology, with a significant increase only observed in amyloid positive MCI cases but not in amyloid negative ones. These findings further support our idea that microglial responses are driven very early during disease progression by seeding of amyloid (Morenas-Rodriguez, Lancet Neurol, 2022). Finally, the biological functions of our top hits further emphasize that lipid dysmetabolism may be a common feature of neurodegenerative disorders.

The following experts could review our manuscript:

Alison Goate ([alison.goate@mssm.edu](mailto:alison.goate@mssm.edu)) - AD risk genes and search for biomarkers

Annakaisa Haapasalo ([annakaisa.haapasalo@uef.fi](mailto:annakaisa.haapasalo@uef.fi)) - Human iPSC derived models, translation in FTD and AD; biomarkers in FTD and AD

Tarja Malm ([tarja.malm@uef.fi](mailto:tarja.malm@uef.fi)) - Neuronflammation; translation, biomarker studies

Eric McDade ([ericmcdade@wustl.edu](mailto:ericmcdade@wustl.edu)) - Heads the biomarker studies in DIAN

Douglas Galasko ([dgalasko@ucsd.edu](mailto:dgalasko@ucsd.edu)) - Specialist for memory disorders and diagnostic testing

Beth Stevens ([beth.stevens@childrens.harvard.edu](mailto:beth.stevens@childrens.harvard.edu)) - Microglial dysfunction AD and related disorders; overlooks the entire field from basic research to translation

Tony Wyss-Coray ([twc@stanford.edu](mailto:twc@stanford.edu)) - Conducts some of the most innovative biomarker studies

Due to a conflict of interest, we would like to exclude Marco Colonna (Washington University School of Medicine in St. Louis) from reviewing our manuscript.

Sincerely,

Christian Haass & Ida Pesämaa

All authors have read and approved the manuscript for publication. The content of this manuscript has not been published, nor is it currently under consideration by any other journal.

**Declaration of interests**

CH collaborates with Denali Therapeutics. HZ has served at scientific advisory boards and/or as a consultant for Abbvie, Acumen, Alector, Alzinova, ALZPath, Annexon, Apellis, Artery Therapeutics, AZTherapies, CogRx, Denali, Eisai, Nervgen, Novo Nordisk, Optoceutics, Passage Bio, Pinteon Therapeutics, Prothena, Red Abbey Labs, reMYND, Roche, Samumed, Siemens Healthineers, Triplet Therapeutics, and Wave, has given lectures in symposia sponsored by Cellectricon, Fujirebio, Alzecure, Biogen, and Roche, and is a co-founder of Brain Biomarker Solutions in Gothenburg AB (BBS), which is a part of the GU Ventures Incubator Program (outside submitted work).

This work was supported by the Deutsche Forschungsgemeinschaft (DFG, German Research Foundation) under Germany’s Excellence Strategy within the framework of the Munich Cluster for Systems Neurology (EXC 2145 SyNergy – ID 390857198 to CH, SFL and DP) and a Koselleck Project HA1737/16-1 (to CH). Further funding came from Alzheimer’s Association (to CH and DP), Vascular Dementia Research Foundation (to DP), and the donors of ADR AD2019604S, a program of the BrightFocus Foundation (to DP). HZ is a Wallenberg Scholar supported by grants from the Swedish Research Council (#2022-01018), the European Union’s Horizon Europe research and innovation programme under grant agreement No 101053962, Swedish State Support for Clinical Research (#ALFGBG-71320), the Alzheimer Drug Discovery Foundation (ADDF), USA (#201809-2016862), the AD Strategic Fund and the Alzheimer's Association (#ADSF-21-831376-C, #ADSF-21-831381-C, and #ADSF-21-831377-C), the Bluefield Project, the Olav Thon Foundation, the Erling-Persson Family Foundation, Stiftelsen för Gamla Tjänarinnor, Hjärnfonden, Sweden (#FO2022-0270), the European Union’s Horizon 2020 research and innovation programme under the Marie Skłodowska-Curie grant agreement No 860197 (MIRIADE), the European Union Joint Programme – Neurodegenerative Disease Research (JPND2021-00694), and the UK Dementia Research Institute at UCL (UKDRI-1003).

The ARTFL-LEFFTDS Longitudinal Frontotemporal Lobar Degeneration (ALLFTD) study (supported through a National Institute of Aging (NIA) and National Institute of Neurological Disorders and Stroke (NINDS) grant U19AG063911.
